# Ethnic education policy awareness and national community identity among ethnic minority college students in China: the dual mediating roles of policy fairness perception and ethnic identity

**DOI:** 10.3389/fpsyg.2026.1862762

**Published:** 2026-08-11

**Authors:** Xuchuan Lei, Lihao Wang

**Affiliations:** 1 School of Public Administration, Southwest Jiaotong University, Chengdu, China; 2 Public Opinion Survey Center, Southwest Jiaotong University, Chengdu, China; 3 Asian Political Culture and Public Governance Center, Southwest Jiaotong University, Chengdu, China

**Keywords:** ethnic education policy, policy fairness perception, ethnic identity, interethnic contact, sense of Chinese national community, policy feedback theory, social identity theory, China

## Abstract

**Introduction:**

How ethnic education policy awareness relates to the sense of Chinese national community (SCNC)—reflecting ethnic minority individuals’ psychological identification with China as a unified multiethnic polity—remains underexamined in Chinese higher education. Integrating policy feedback theory, social identity theory, and intergroup contact theory, this study examines the parallel mediating roles of policy fairness perception and ethnic identity, as well as the moderating role of interethnic contact depth.

**Methods:**

A cross-sectional survey was conducted among 360 ethnic minority college students in China (68.3% female; aged 18 years and above, including adult learners; participants from the Miao, Tujia, Yi, Tibetan, and other ethnic groups). The data were analyzed using structural equation modeling with 5,000 bias-corrected bootstrap resamples and conditional process analysis.

**Results:**

Policy awareness had no significant direct association with SCNC, whereas two significant parallel indirect associations were identified. The indirect effect through policy fairness perception was 0.078 (95% CI [0.043, 0.202]), and the indirect effect through ethnic identity was 0.144 (95% CI [0.059, 0.288]); the two indirect effects did not differ significantly in magnitude. Although the interaction between ethnic identity and interethnic contact depth was negative at the path level, the index of moderated mediation was not statistically significant and should therefore be interpreted as exploratory. Gender and regional heterogeneity in pathway strength were also observed.

**Discussion:**

The findings suggest that ethnic education policy awareness is associated with SCNC primarily through fairness evaluations and ethnic identity rather than through a direct pathway. Policy communication transparency, ethnic cultural programming, and structured interethnic contact may represent complementary institutional approaches to cultivating national community identification. Longitudinal and experimental research is needed to establish temporal ordering and further examine the moderating role of interethnic contact.

## 1 Introduction

China recognizes 55 officially designated ethnic minority groups comprising approximately 8.5% of the total population and inhabiting roughly 60% of the national territory (Hansen, 2011; Postiglione, 2013). Since the 1980s, the state has implemented a broad portfolio of preferential education policies for ethnic minorities—including priority university admission, tuition subsidies, preparatory education programs, and bilingual instruction support—with the dual aims of narrowing educational opportunity gaps and cultivating minority students’ identification with the Chinese national community. Despite the scope of these interventions, research has consistently documented a gap between policy exposure and identity formation: heightened policy awareness does not straightforwardly generate stronger national community identification (Campbell, 2012; Mettler and Soss, 2004). This transmission gap between policy input and identity outcome motivates the present inquiry.

At the center of this study is the sense of Chinese national community (SCNC; Chinese: zhōnghuá mínzú gòngtóngtǐ yìshí). Grounded in China’s widely recognized “plurality within unity” framework of multiethnic national identity, SCNC reflects ethnic minority individuals’ psychological identification with China as a unified multiethnic polity. It encompasses cognitive recognition of China’s multiethnic historical community, emotional attachment to the national community as a superordinate group, and behavioral commitment to national harmony and development Chen and Xue, 2021; Ma et al., 2024). Conceptually, SCNC shares theoretical affinities with superordinate group identification in Western social psychology (Gaertner and Dovidio, 2014; Huo et al., 1996) and with the broader literature on civic national identity (Brewer, 1991; Verkuyten and Yildiz, 2007), though it is embedded in a distinctly Chinese sociopolitical context. It is important to distinguish SCNC from related but conceptually distinct constructs. National identity in the Western literature typically refers to identification with one’s nation-state as a political community (Brewer, 1991; Verkuyten and Yildiz, 2007), while civic identity emphasizes rights, duties, and participation in democratic institutions (Huo et al., 1996). SCNC is theoretically related to superordinate group identification but is embedded in China’s distinctive plurality-within-unity framework, which conceptualizes ethnic and national identities as mutually compatible rather than competing. Ethnic identity, by contrast, refers to individuals’ affective attachment to and behavioral engagement with their own ethnic group’s cultural heritage (Phinney, 1992; Verkuyten, 2018)—a subgroup-level construct that may coexist with superordinate national identification.

Policy feedback theory (Campbell, 2012; Mettler and Soss, 2004; Pierson, 1993) provides the first explanatory pathway. The framework argues that public policies shape citizens’ attitudes and identities through two complementary mechanisms: resource effects, in which material benefits alter recipients’ social position, and interpretive effects, in which the symbolic content of policy signals shapes political cognition. Applied to ethnic education policy, students who possess clear and comprehensive policy awareness are more likely to evaluate these policies in structured, system-oriented terms—judging whether the state has implemented them transparently and equitably. Policy fairness perception (PFP), encompassing both procedural and distributive dimensions, represents this evaluative response and has been identified as a key mechanism through which policy awareness fosters broader national identification (Guo, 2023; Lind and Tyler, 1988; Tyler, 1990). It is important to note that institutional trust represents a broader evaluative orientation toward governmental and educational institutions as competent and benevolent actors, while PFP is the more proximate, policy-specific psychological mechanism through which such trust is enacted in the context of preferential education policy. The present study measures and tests PFP as the operational mediator, consistent with the procedural justice tradition (Lind and Tyler, 1988; Tyler, 1990). We focus on PFP and ethnic identity as mediators because they represent the most theoretically proximate psychological mechanisms in this context. The empirical literature has most consistently implicated fairness judgments and identity processes as the primary channels of policy influence (Guo, 2023; Mettler and Soss, 2004). This rationale is also consistent with system justification theory (Jost et al., 2004), which holds that individuals evaluate institutional arrangements through fairness lenses that subsequently shape broader system identification.

Social identity theory (Tajfel and Turner, 2004) provides the second explanatory pathway. This framework holds that individuals derive a significant portion of their self-concept from group memberships, and that institutional environments supply the reference frames through which self-categorization operates. Ethnic education policies, as institutionalized expressions of state recognition for ethnic cultures, transmit a symbolic message that minority identities are valued by the national polity. When students clearly understand and internalize this message, their attachment to their own ethnic cultural heritage—ethnic identity (EI)—is strengthened. In turn, robust ethnic identity need not compete with superordinate national identification: when ethnic and national identities are perceived as culturally compatible, higher ethnic identity may facilitate the development of national community belonging (Brewer, 1991; Verkuyten and Yildiz, 2007), consistent with China’s plurality-within-unity conceptualization of multiethnic national identity.

A third theoretical lens addresses the conditions under which the ethnic identity pathway operates. Intergroup contact theory (Allport, 1954; Pettigrew and Tropp, 2006) proposes that under conditions of equal status, cooperative goals, and institutional support, positive contact between members of different groups reduces prejudice and promotes intergroup integration. Particularly relevant is Pettigrew’s (1998) recategorization mechanism: deep intergroup contact may enable individuals to transcend original group boundaries and develop identification with a more inclusive superordinate group (Gaertner and Dovidio, 2014). In the multiethnic university context, depth of interethnic contact (IEC) may moderate the relationship between ethnic identity and national community identification—either by amplifying the ethnic identity pathway (the traditional amplifier prediction) or by enabling a more direct activation of superordinate national belonging that reduces reliance on ethnic identity as an intermediary (the recategorization prediction). The present study holds this direction open as an empirical question.

The three theoretical lenses are integrated as follows. Policy feedback theory explains the input stage: policy awareness triggers evaluative cognitions about institutional fairness. Social identity theory explains the identity processing stage: these cognitions shape ethnic identity, which connects to superordinate national identification. Intergroup contact theory addresses the boundary condition: contact depth modulates how effectively ethnic identity mediates national belonging. Each theory thus operates at a distinct causal stage, making their integration complementary rather than redundant. Despite this theoretical richness, three specific empirical gaps persist. First, no prior study has simultaneously tested the policy fairness and social identity mechanisms within a single unified framework, preventing direct comparison of their relative contributions. Second, the boundary conditions of the ethnic identity pathway—particularly the role of interethnic contact depth—have received no empirical attention in Chinese higher education. Third, moderated parallel mediation, which enables simultaneous testing of competing mechanisms and their differential sensitivity to contextual moderators, has not been applied to this question. The present study addresses these gaps with the following hypotheses:

*H1a*: Ethnic education policy awareness is positively associated with policy fairness perception.

*H1b*: Policy fairness perception is positively associated with the sense of Chinese national community.

*H1*: Ethnic education policy awareness exerts a significant positive indirect effect on the sense of Chinese national community through policy fairness perception (policy fairness pathway).

*H2a*: Ethnic education policy awareness is positively associated with ethnic identity.

*H2b*: Ethnic identity is positively associated with the sense of Chinese national community.

*H2*: Ethnic education policy awareness exerts a significant positive indirect effect on the sense of Chinese national community through ethnic identity (social identity pathway).

*H3:* (Exploratory): Depth of interethnic contact moderates the association between ethnic identity and the sense of Chinese national community; the specific direction is held open pending empirical testing.

**FIGURE 1:**
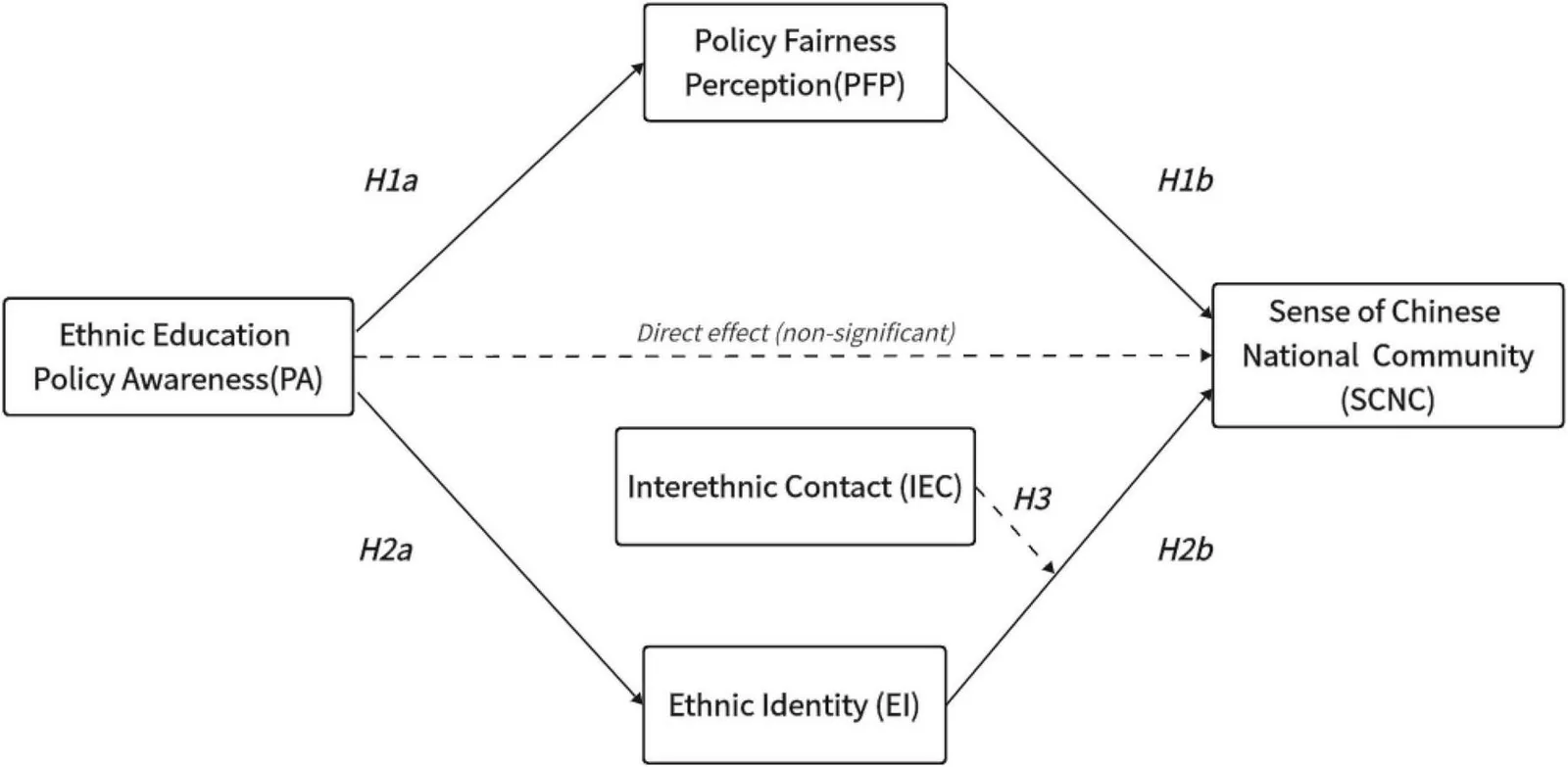
Conceptual model of the moderated parallel mediation. Dashed lines denote the moderating effect of interethnic contact on the ethnic identity pathway. PA, policy awareness; PFP, policy fairness perception; EI, ethnic identity; IEC, interethnic contact; SCNC, sense of Chinese national community.

The proposed conceptual model is presented in Figure 1.

## 2 Materials and methods

### 2.1 Participants and procedure

An online cross-sectional survey was administered in March 2025 via Credamo (Jiànshù), a third-party Chinese survey platform that implements multi-layered automated quality control. The platform employs algorithms to detect and exclude aberrant response patterns—including completion times below engagement thresholds, straight-line responding, and repeated identical response sequences—providing a systematic first-stage filter for data quality prior to manual cleaning. Eligibility required participants to be currently enrolled ethnic minority college students aged 18 years or above. All participants provided written electronic informed consent and were assured of the voluntary and anonymous nature of the study. After data cleaning, a total of 360 valid responses were retained. The age distribution of the sample warrants explicit clarification. While 52.8% of participants fall within the 18–26 age range typical of conventional full-time undergraduates, 42.5% are aged 27–35 and 4.7% are older than 35. This distribution reflects the inclusion of students enrolled through China’s adult higher education system (成人高等教育), a formally recognized pathway allowing working adults to pursue accredited higher education qualifications while maintaining formal student status. All participants hold formal enrollment status at Chinese higher education institutions and fall within the broader Chinese usage of the term college students (大学生). In terms of educational stage: 7.8% are in preparatory programs, 72.2% are undergraduates, 19.2% are master’s students, and 0.8% are doctoral students or above. Future research should explicitly stratify by enrollment type to examine whether findings replicate across traditional and non-traditional student subgroups. Furthermore, adult higher education students and conventional full-time students may differ systematically in work experience, political socialization, and social networks, all of which could influence policy awareness, ethnic identity, and interethnic contact patterns. Subgroup comparisons between these populations in future research would strengthen the generalizability of the present findings.

Demographic characteristics are summarized in Table 1. The sample comprised 246 females (68.3%) and 114 males (31.7%), was predominantly aged 23–35 years, and included students from Miao, Tujia, Yi, Tibetan, and other minority groups. Participants were drawn from undergraduate and graduate programs at multiple institutions, with the majority reporting that they had benefited from at least one preferential ethnic minority education policy.

**TABLE 1:** Demographic characteristics of participants.

Variable	Category	*n*	%
Gender	Male	114	31.7
	Female	246	68.3
Age (years)	18–22	85	23.6
	23–26	105	29.2
	27–35	153	42.5
	> 35	17	4.7
Ethnic group	Miao	111	30.8
	Tujia	49	13.6
	Yi	28	7.8
	Tibetan	19	5.3
	Other minorities	153	42.5
Region of origin	Southwest China	224	62.2
	Other regions	136	37.8
Education	Below undergraduate	28	7.8
	Undergraduate	260	72.2
	Master’s	69	19.2
	Doctoral or above	3	0.8
Policy beneficiary	Yes	331	91.9
	No	29	8.1

*N* = 360.

### 2.2 Measures

All constructs were measured using Likert-type items rated on a 5-point scale (1 = strongly disagree, 5 = strongly agree). Composite scores (mean of items) were used in all analyses. Given the pronounced negative skewness of all scale distributions (skewness range: −1.39 to −2.44), internal consistency was assessed using ordinal alpha based on polychoric correlations, which provides more accurate reliability estimates for non-normally distributed ordinal data than Pearson-based Cronbach’s alpha (Gadermann et al., 2012). All reliability coefficients reported below are ordinal alphas. The complete English and Chinese wording of all scale items is provided in Supplementary Appendix A.

#### 2.2.1 Policy awareness (PA)

Three items assessed participants’ familiarity with and knowledge of preferential ethnic education policies at their institution (e.g., “I am familiar with the preferential admission policies for ethnic minority students at my university”). Items were adapted from Wang (2023) in accordance with the operationalization of cognitive policy effects in policy feedback theory (Mettler and Soss, 2004). Ordinal alpha = 0.76.

#### 2.2.2 Policy fairness perception (PFP)

Three items assessed procedural fairness (transparency and consistency of policy implementation) and distributive fairness (equitable allocation of educational resources), adapted from Lind and Tyler (1988) and Tyler (1990) with context-specific modifications validated for ethnic minority college students by Guo (2023). Ordinal alpha = 0.72.

#### 2.2.3 Ethnic identity (EI)

Five items assessed affective attachment to participants’ ethnic cultural heritage and behavioral engagement with ethnic traditions, adapted from Phinney, (1992) Multigroup Ethnic Identity Measure (MEIM) with Chinese validation by Chen et al. (2016). Ordinal alpha = 0.74.

#### 2.2.4 Interethnic contact (IEC)

Three items measured depth of interethnic contact, focusing on proactive cross-ethnic engagement: collaborative participation in joint tasks or community activities, active curiosity about others’ cultural backgrounds, and constructive communication when cultural misunderstandings arise. This operationalization captures the behavioral initiative and substantive depth of interethnic contact emphasized by Pettigrew and Tropp (2006), and is conceptually distinct from contact frequency, which refers to the quantity rather than the quality or depth of cross-ethnic interaction (Brown and Hewstone, 2005). Ordinal alpha = 0.76.

#### 2.2.5 Sense of Chinese national community (SCNC)

Four items assessed emotional belonging to China as a multiethnic nation, alignment with national community values, and acceptance of multicultural coexistence, adapted from Chen and Xue, 2021 and the three-dimensional framework developed by Ma et al. (2024). Ordinal alpha = 0.71.

### 2.3 Analytical strategy

Analyses proceeded in four stages. First, confirmatory factor analysis (CFA) was conducted in AMOS 28.0 to evaluate the measurement model and to establish convergent and discriminant validity following criteria recommended by Fornell and Larcker (1981), Hair et al. (2019), and Hu and Bentler (1999). Second, Harman’s single-factor test and a competing single-factor model were used to assess the extent of common method bias. Third, the parallel mediation model was estimated via structural equation modeling (SEM) in AMOS, with indirect effects tested using bias-corrected bootstrapped confidence intervals based on 5,000 resamples (Preacher and Hayes, 2008). Fourth, moderated mediation was examined using Hayes (2017) PROCESS macro (Model 14) in SPSS, with continuous predictors mean-centered before computing interaction terms. Gender, age, education level, and region of origin were included as covariates throughout.

## 3 Results

### 3.1 Descriptive statistics and correlations

Descriptive statistics, composite reliabilities, and inter-construct correlations are summarized in Table 2. All constructs demonstrated acceptable internal consistency. Policy awareness showed positive correlations with both mediators, and both mediators were positively correlated with national community identification, providing preliminary support for the hypothesized mediation structure. The pattern of inter-construct correlations indicated that all five constructs are empirically distinguishable from one another.

**TABLE 2:** Descriptive statistics, composite reliabilities, and correlations among study variables.

Variable	*M*	*SD*	CR	1	2	3	4	5
1. PA	4.38	0.55	0.76	0.714	0.679	0.616	0.714	0.648
2. PFP	4.33	0.46	0.72	0.490				
3. EI	4.48	0.35	0.74	0.387	0.363			
4. IEC	4.38	0.46	0.76	0.375	0.290	0.444		
5. SCNC	4.62	0.32	0.71	0.212	0.288	0.425	0.212	

Diagonal entries are the square root of the average variance extracted (AVE). PA, policy awareness; PFP, policy fairness perception; EI, ethnic identity; IEC, interethnic contact; SCNC, sense of Chinese national community. *N* = 360.

### 3.2 Measurement model

CFA was conducted to evaluate the five-factor measurement structure. As shown in Tables 3–5, the five-factor model demonstrated acceptable fit to the data and substantially outperformed the alternative four-factor and one-factor models. All standardized factor loadings were statistically significant and within an acceptable range, supporting item reliability. Convergent validity was supported by composite reliability (CR) values exceeding the recommended 0.70 threshold for all constructs. Average variance extracted (AVE) values for policy awareness and interethnic contact met the conventional 0.50 criterion; values for the remaining three constructs were marginally below this threshold. Given that CR values remained adequate for all constructs, this marginal shortfall is attributable to the multidimensional nature of these constructs and a ceiling effect in student responding, and represents a measurement limitation that should be interpreted with appropriate caution (Fornell and Larcker, 1981). Preliminary evidence for discriminant validity was provided using the Fornell-Larcker criterion: for each construct, the square root of its AVE exceeded its highest correlation with any other construct (Table 2).

**TABLE 3:** Confirmatory factor analysis: standardized loadings, composite reliability, and average variance extracted.

Construct	Item	Std. Loading	*t*-value	CR	AVE
PA	1	0.563	Fixed	0.76	0.51
	2	0.672	8.934		
	3	0.724	9.387		
PFP	4	0.628	Fixed	0.72	0.46
	5	0.524	7.641		
	6	0.673	8.209		
EI	7	0.584	Fixed	0.74	0.38
	8	0.612	8.117		
	9	0.473	6.583		
	10	0.642	8.476		
	11	0.531	7.214		
IEC	12	0.634	Fixed	0.76	0.51
	13	0.612	8.654		
	14	0.674	9.012		
SCNC	15	0.652	Fixed	0.71	0.39
	16	0.483	7.138		
	17	0.591	8.014		
	18	0.665	8.972		

All loadings significant at *p* < 0.001. “Fixed” denotes the reference indicator constrained to unity for model identification; *t*-values are not applicable for fixed indicators. Item numbers are assigned consecutively across constructs.

**TABLE 4:** alternative measurement model comparisons.

Model	χ^2^	df	Δχ^2^	Δdf	*p*	ΔCFI
Five-factor (hypothesized)	359.12	125	–	–	–	–
Four-factor (EI + SCNC merged)	375.13	129	16.01	4	0.003	−0.014
One-factor	427.51	135	68.38	10	< 0.001	−0.069

Δχ^2^ and ΔCFI are relative to the five-factor hypothesized model. CFI values are from AMOS using maximum likelihood estimation. Estimation based on N = 360.

**TABLE 5:** Fit indices for the measurement and structural models.

Model	χ^2^/df	CFI	TLI	IFI	RMSEA	SRMR
Single-factor model	11.247	0.487	0.467	0.489	0.173	0.178
Five-factor measurement model	1.893	0.943	0.934	0.944	0.050	0.059
Parallel mediation SEM	1.927	0.937	0.928	0.938	0.051	0.063

Recommended thresholds: χ^2^/df < 3, CFI/TLI/IFI > 0.90, RMSEA < 0.08, SRMR < 0.08 (Hair et al., 2019; Hu and Bentler, 1999).

### 3.3 Common method bias assessment

Three complementary procedures were used to assess common method bias. First, Harman’s single-factor test indicated that the first unrotated factor accounted for less than 40% of total variance. Second, a single-factor CFA model fit the data extremely poorly relative to the five-factor model (ΔCFI = 0.456, Δχ^2^ = 68.385, Δdf = 10, *p* < 0.001), providing structural evidence that the five constructs are empirically distinguishable (Podsakoff et al., 2003). Third, a marker variable procedure was conducted following Lindell and Whitney (2001). An item theoretically unrelated to the core constructs was selected as the marker variable: “If negotiation fails to resolve issues related to ethnic rights, I would support taking stronger measures to protect those rights” ($\bar{r}$ = 0.090 with all five composites, max *r* = 0.115). Key path coefficients were re-estimated controlling for the marker variable alongside original covariates. All path coefficients remained substantively unchanged after this control (maximum change < 0.01 across all five key paths), providing additional evidence against severe common method variance inflation. We acknowledge nonetheless that these procedures provide convergent rather than conclusive evidence, as all five constructs rely on single-source self-report data collected in a single session.

### 3.4 Parallel mediation

**FIGURE 2:**
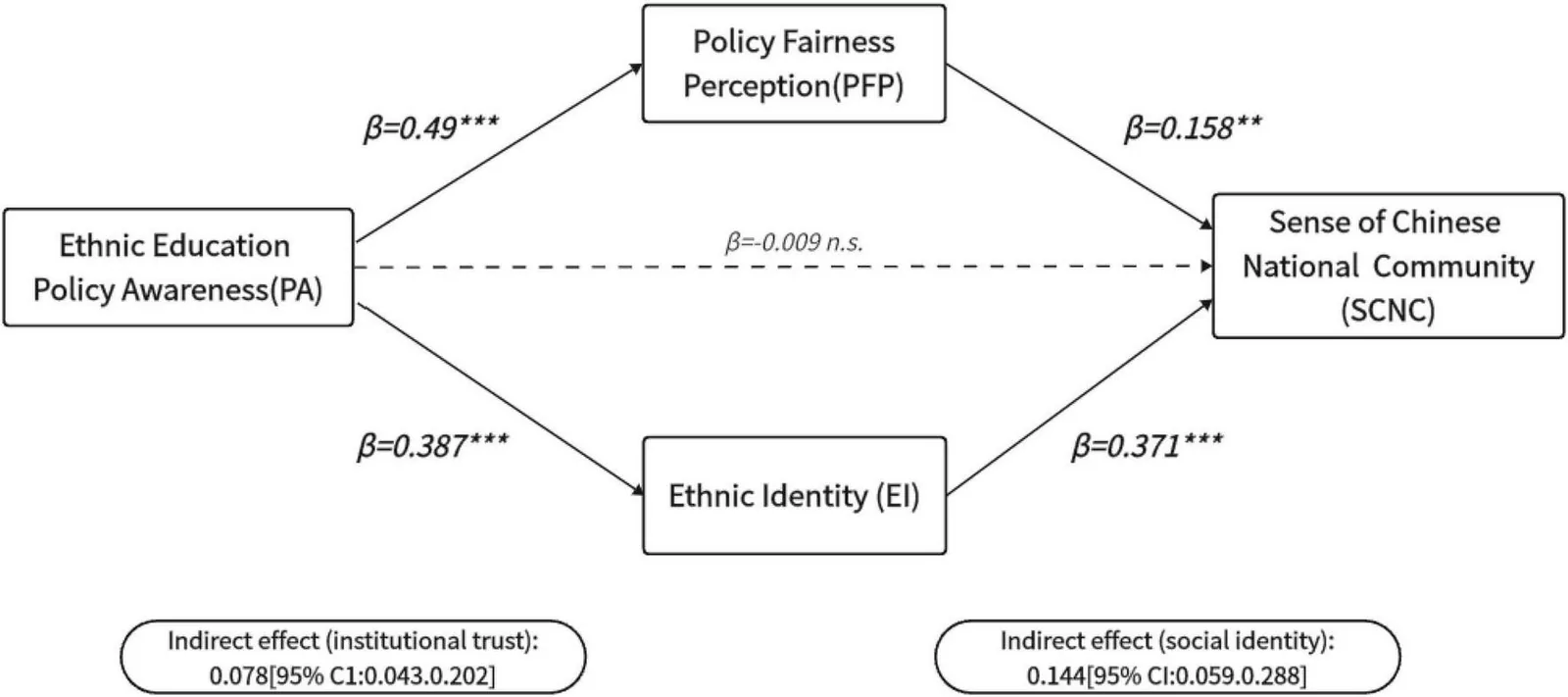
Standardized path coefficients from the parallel mediation SEM. Solid lines indicate significant paths; the dashed line indicates the non-significant direct effect. **P < 0.01; ***P < 0.001; n.s., not significant.

A parallel mediation model was estimated via SEM, with policy awareness as the independent variable, policy fairness perception and ethnic identity as simultaneous mediators, and national community identification as the outcome, controlling for gender, age, education level, and region of origin. The model fit was acceptable (Table 5). As shown in Table 6 and Figure 2, policy awareness was significantly and positively associated with both mediators, supporting H1a and H2a. Both policy fairness perception and ethnic identity were in turn significant positive correlates of national community identification, supporting H1b and H2b. The direct effect of policy awareness on national community identification was not significant (β = −0.009, *p* = 0.871), indicating that its association with national community identification is statistically mediated through the two psychological pathways. The near-zero negative sign is consistent with the suppression pattern characteristic of full mediation, in which the covariance between predictor and outcome is entirely absorbed by the mediating variables (MacKinnon et al., 2000). This pattern is also theoretically coherent within the present framework: policy feedback theory does not predict that mere awareness of a policy is directly linked to identification; rather, it holds that awareness is associated with identification indirectly by first altering citizens’ evaluative judgments and identity-relevant cognitions (Mettler and Soss, 2004; Pierson, 1993). Because the study employs a cross-sectional design, the path coefficients reported here reflect statistical associations consistent with the hypothesized causal model rather than established causal relationships; the directionality of effects cannot be confirmed without longitudinal or experimental evidence.

**TABLE 6:** Standardized path coefficients from structural equation modeling.

Path	β	*SE*	*t*	*p*
PA to PFP	0.490	0.046	10.640	< 0.001
PA to EI	0.387	0.049	7.929	< 0.001
PA to SCNC (direct)	−0.009	0.056	−0.163	0.871
PFP to SCNC	0.158	0.056	2.842	0.005
EI to SCNC	0.371	0.053	7.062	< 0.001

Covariates (gender, age, education, region of origin) were included but are not listed. *N* = 360.

Bootstrap-based tests of indirect effects are reported in Table 7. Confidence intervals for both indirect pathways excluded zero, supporting H1 (the policy fairness pathway) and H2 (the social identity pathway). The confidence interval for the direct effect included zero, corroborating full parallel mediation. Importantly, the two indirect effects did not differ significantly from each other in magnitude, indicating that policy fairness perception and ethnic identity make comparably substantial contributions to the statistical transmission of policy awareness’s association with national community identification. In terms of practical magnitude, the indirect effects via the two pathways (β = 0.078 and β = 0.144) fall in the small-to-moderate range by conventional standards (Cohen, 2013), consistent with typical effect sizes reported in policy feedback and identity research.

**TABLE 7:** Bootstrap mediation analysis: indirect effects (5,000 resamples, bias-corrected confidence intervals).

Effect	*b*	*SE*	95% CI LL	95% CI UL
Policy fairness pathway (PA, via PFP, to SCNC)	0.078	0.033	0.043	0.202
Social identity pathway (PA, via EI, to SCNC)	0.144	0.048	0.059	0.288
Direct effect (PA to SCNC)	−0.009	0.071	−0.136	0.142
Total indirect effect (PA to SCNC)	0.221	0.062	0.147	0.423

Confidence intervals not containing zero indicate statistically significant effects. LL, lower limit; UL, upper limit. *N* = 360.

### 3.5 Moderated mediation

To examine whether depth of interethnic contact moderated the ethnic identity pathway, Hayes (2017) PROCESS macro (Model 14) was applied with policy awareness as the independent variable, ethnic identity as the mediator, interethnic contact as the moderator, and national community identification as the outcome. Predictors were mean-centered before computing the interaction term. Results are presented in Table 8.

**TABLE 8:** Moderated mediation analysis: interaction effect, simple slopes, and conditional indirect effects.

Predictor	b	*SE*	*t*	95% CI LL	95% CI UL
Moderation analysis (outcome: SCNC)					
EI (main effect)	0.368	0.063	5.841	0.244	0.492
IEC (main effect)	−0.017	0.054	−0.315	−0.123	0.089
EI × IEC	−0.043	0.021	−2.002	−0.084	−0.002
Simple slope analysis					
Low IEC (−1 SD): EI predicting SCNC	0.411	0.068	6.044	0.277	0.545
High IEC (+1 SD): EI predicting SCNC	0.325	0.061	5.328	0.205	0.445
Conditional indirect effects of PA on SCNC via EI					
Low interethnic contact (−1 SD)	0.159	0.061	–	0.049	0.279
High interethnic contact (+1 SD)	0.126	0.059	–	0.051	0.247
Index of moderated mediation (IMM)	−0.033	0.025	–	−0.172	0.131

EI and IEC were mean-centered before computing the 60. interaction term. Bootstrap resamples = 5,000; 95% confidence intervals. – = not applicable. *N* = 3.

**FIGURE 3:**
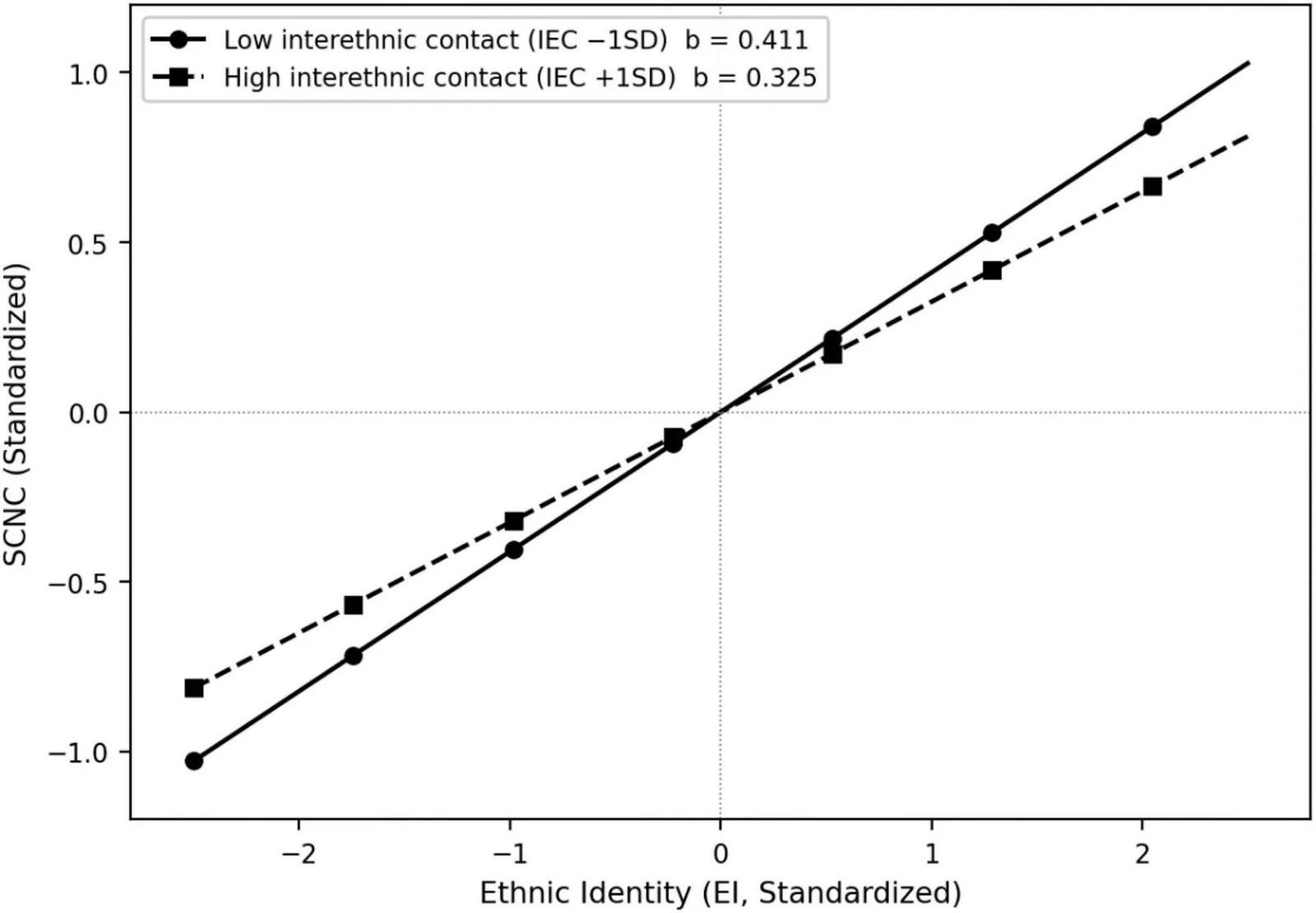
Simple slope analysis illustrating the moderating role of interethnic contact (IEC) on the association between ethnic identity (EI) and sense of Chinese national community (SCNC). Both slopes are statistically significant. Low IEC, - 1 SD; High IEC, +1 SD.

The interaction between ethnic identity and interethnic contact was statistically significant and negative in direction. Contrary to the traditional amplifier prediction, students who engaged more frequently in deep cross-ethnic contact showed a weaker association between ethnic identity and national community identification than students with less interethnic contact. Simple slope analysis (Table 8 and Figure 3) confirmed that the ethnic identity pathway remained significant under both low and high interethnic contact conditions, but was comparatively stronger when interethnic contact was limited.

Conditional indirect effect analyses showed that the indirect effect of policy awareness on national community identification through ethnic identity was statistically significant under both low and high interethnic contact conditions (Table 8). However, the index of moderated mediation (IMM) did not attain statistical significance (IMM = −0.033, 95% CI [−0.172, 0.131]), indicating that the overall conditional indirect effect cannot be reliably distinguished from zero across levels of interethnic contact. H3 is therefore not supported at the level of the overall moderated mediation effect. The EI × IEC interaction term was significant at the path level (β = −0.043, *p* = 0.045), and this path-level observation is noted as a preliminary and exploratory finding only. One possible explanation for the non-significant IMM is that the restricted variance of the SCNC outcome reduced statistical power to detect a conditional indirect effect; however, we acknowledge this explanation is speculative in the absence of formal power or sensitivity analysis, and we cannot rule out the possibility that the moderated mediation effect is genuinely weak or absent in this population. The non-significant IMM should be treated as the primary finding for H3. The path-level interaction is noted only as a preliminary observation requiring replication with larger samples, more sensitive outcome measures, and ideally an experimental or longitudinal design before any substantive conclusions can be drawn.

### 3.6 Heterogeneity analyses

The following multi-group analyses are presented as exploratory findings. Formal tests of metric and scalar measurement invariance were not conducted; configural invariance analyses in AMOS confirmed that the five-factor structure is broadly recoverable across all subgroups (gender: male CFI = 0.890, RMSEA = 0.089; female CFI = 0.930, RMSEA = 0.067; region: Southwest CFI = 0.830, RMSEA = 0.092; non-Southwest CFI = 0.900, RMSEA = 0.044). The Southwest subgroup CFI of 0.830 falls slightly below the conventional 0.90 threshold, which is attributable to more pronounced ceiling effects in that subsample (Chen, 2007). Readers should interpret between-group comparisons of path coefficients with appropriate caution in the absence of formal invariance testing. Multi-group SEM with gender and region of origin as grouping variables revealed the following exploratory patterns. Gender differences (n male = 114, n female = 246): Multi-group analysis showed that the pathway from policy awareness to policy fairness perception was significantly stronger among male participants (β = 0.665) than among female participants (β = 0.392), with the difference reaching statistical significance (Δβ = −0.273, z = −3.08, *p* = 0.002). By contrast, the pathway from policy fairness perception to national community identification (β male = .250 vs. β female = .301) and the pathway from ethnic identity to national community identification (β male = 0.457 vs. β female = 0.414) did not differ significantly across gender groups (both *p* > 0.60). These patterns suggest that male and female students differ in the degree to which policy awareness is associated with fairness perceptions, but that once policy fairness perception or ethnic identity is activated, both groups show comparable associations between these psychological states and national community identification.

Regional differences (n Southwest = 224, n non-Southwest = 136): Regional differences were more pronounced. The pathway from policy fairness perception to national community identification was significantly weaker in the non-Southwest group (β = 0.153) than in the Southwest group (β = 0.448; Δβ = −0.295, *z* = −3.08, *p* = 0.002). Similarly, the pathway from ethnic identity to national community identification was significantly weaker among non-Southwest participants (β = 0.156) than among Southwest participants (β = 0.684; Δβ = −0.528, *z* = −5.93, *p* < 0.001). In addition, the pathway from policy awareness to ethnic identity was significantly stronger in the Southwest group (β = 0.533) than in the non-Southwest group (β = 0.251; Δβ = −0.282, *z* = −2.83, *p* = 0.005). This pattern suggests that in regions with a high concentration of ethnic minority cultures, the full chain from policy awareness through ethnic identity to national community identification operates with considerably greater strength; outside these culturally dense contexts, both terminal pathways are markedly attenuated, indicating that the dual-pathway model, while robust in its overall structure, varies substantially in intensity as a function of regional cultural context.

## 4 Discussion

### 4.1 Summary of key findings

This study examined how ethnic education policy awareness shapes the sense of Chinese national community among ethnic minority college students through a moderated parallel mediation framework. Three substantive findings merit discussion.

First, policy awareness exerted no significant direct influence on national community identification; its effects were fully transmitted through two parallel psychological pathways of statistically equivalent explanatory weight. This finding of full parallel mediation challenges accounts that treat policy communication as a straightforward driver of national identification, and underscores that the relationship is more complex and psychologically mediated than is often assumed. The equivalence of the two pathways further suggests that efforts to foster national community identification through either enhanced perceptions of institutional fairness or reinforced ethnic cultural pride are likely to prove equally productive.

Second, a negative interaction between ethnic identity and interethnic contact was observed at the path level: greater interethnic contact was associated with a relatively weaker association between ethnic identity and national community identification. We acknowledge that the non-significant index of moderated mediation (IMM) constitutes an important limitation of this analysis. The path-level interaction, while statistically significant, is insufficient to confirm moderated mediation, and we caution readers against over-interpreting this finding. One possible explanation is that intensive cross-ethnic interaction may activate national belonging more directly, reducing reliance on ethnic identity as an intermediary—a pattern consistent with recategorization theory (Pettigrew, 1998) and the common in-group identity model (Gaertner and Dovidio, 2014). Beyond recategorization, contact-induced decategorization (Brewer, 1991) may temporarily suppress ethnic salience during intergroup interaction. However, we cannot rule out the possibility that the moderated mediation effect is genuinely weak or absent in this population. This path-level observation warrants replication with larger samples, more sensitive outcome measures, and ideally an experimental or longitudinal design before any substantive conclusions are drawn.

Third, gender and regional heterogeneity analyses revealed that the dual-pathway model operates with different intensities across demographic subgroups, though these are exploratory findings to be interpreted with caution given the absence of formal measurement invariance testing. With respect to gender, male participants showed a stronger association between policy awareness and policy fairness perception, suggesting greater sensitivity to the institutional cognition channel at the input stage, while the terminal pathways from mediators to national community identification were comparable across genders, indicating that the associations between policy fairness perception, ethnic identity, and national belonging are comparably robust across genders. The regional differences were more striking: both the policy fairness pathway and the social identity pathway were substantially stronger among Southwest China participants than among those raised elsewhere, consistent with the interpretation that a culturally dense minority context amplifies the resonance of policy awareness and ethnic identity as correlates of national belonging. These findings underscore the importance of contextual and individual variability in how policy awareness is psychologically processed.

### 4.2 Theoretical implications

The present findings contribute to theory in three respects. First, by simultaneously testing policy fairness perception and social identity mechanisms within a unified parallel mediation framework, this study provides more complete evidence for the transmission gap documented in the policy feedback literature: policy awareness is a necessary but insufficient condition for national community identification, and its association with identification is statistically mediated through distinct psychological channels. The parallel structure reveals that the two mechanisms are complementary rather than mutually exclusive, jointly accounting for the statistical association between policy inputs and identity outputs in ways that single-pathway models cannot capture (Campbell, 2012; Mettler and Soss, 2004). This finding is also consistent with system justification theory (Jost et al., 2004), which holds that individuals evaluate institutional arrangements through fairness lenses that subsequently shape broader system identification. Institutional trust, understood as the broader evaluative orientation toward governmental institutions, is the theoretical backdrop against which the more proximate mechanism of policy fairness perception operates.

The salience of policy fairness perception as a mechanism is consistent with procedural justice theory (Lind and Tyler, 1988; Tyler, 1990), which holds that individuals’ evaluations of institutional processes—more than the material outcomes they receive—shape their identification with and loyalty toward the institutions concerned. In the ethnic education policy context, students who perceive policies as transparently implemented and equitably distributed receive a symbolic message that the state treats their group with procedural respect. This recognition strengthens identification with the national polity as a fair and inclusive institution, thereby activating the broader sense of national community belonging.

Second, by introducing intergroup contact theory into the study of policy-identity linkages, this study identifies a moderating boundary condition that has been largely overlooked in the Chinese ethnic education literature. The finding that interethnic contact attenuates rather than amplifies the ethnic identity pathway extends Pettigrew and Tropp (2006) meta-analytic conclusions to the domain of national community identification, and is consistent with the view that high-quality interethnic interaction constitutes an independent pathway to superordinate identification (Gaertner and Dovidio, 2014; Huo et al., 1996). Future models should therefore treat interethnic contact not merely as a background variable but as an active psychological mechanism with distinct implications for how ethnic and national identities develop in relation to each other.

The finding of full parallel mediation with no significant direct effect is broadly consistent with the interpretive-effects tradition in policy feedback research (Mettler and Soss, 2004; Pierson, 1993), which holds that policy awareness alone is an insufficient condition for identification—what matters is how awareness is cognitively and emotionally processed. The ethnic identity pathway replicates and extends prior Chinese findings on the compatibility of ethnic and national identification (Guo, 2023); Verkuyten and Yildiz, 2007), while the policy fairness pathway adds a dimension that prior single-pathway studies could not isolate. Together, these pathways reflect a more complete picture of how ethnic education policy enters the psychological processes that ultimately produce national community belonging.

Third, the absence of a direct effect of policy awareness on national community identification, combined with full parallel mediation through two distinct psychological channels, clarifies a recurring empirical puzzle in the ethnic education literature. This study does not argue that this pattern is definitively predicted by any single theoretical framework; rather, one plausible interpretation is that it is consistent with the interpretive-effects dimension of policy feedback theory (Pierson, 1993), which holds that policy inputs reshape identification not through direct communication but by first altering the evaluative and identity-relevant cognitions of recipients. Under this reading, the absence of a direct effect is not an anomaly but a theoretically coherent finding indicating that psychological mediation fully accounts for the policy-to-identification link. Alternative interpretations are possible—for instance, the finding may partly reflect ceiling effects or common-method constraints rather than a true absence of direct influence—and future research using experimental or longitudinal designs would be needed to adjudicate among competing explanations. Regardless of the precise mechanism, the practical implication is clear: examining mediating processes, rather than stopping at direct effects, is essential for meaningful policy evaluation research.

Several features of China’s ethnic policy environment warrant noting for comparative purposes. The explicit constitutional recognition of ethnic minority rights, the preferential education policy infrastructure, and the plurality-within-unity ideology that frames ethnic and national identities as complementary rather than competing collectively create an institutional context that is distinct from Western multicultural democracies. In such a context, both the policy fairness and social identity pathways may be particularly salient because the state actively cultivates both dimensions simultaneously through policy design and political education. Whether similar dual pathways operate in assimilationist or pluralist but market-driven educational systems remains an open comparative question. At the same time, the underlying psychological mechanisms—procedural fairness evaluations and identity-based superordinate group membership—are theoretically general and may operate across diverse institutional contexts, suggesting that the dual-pathway model may have broader applicability beyond the Chinese case.

### 4.3 Practical implications

Because both the policy fairness pathway and the social identity pathway are independently significant and of comparable magnitude, institutions seeking to cultivate national community identification would benefit from a dual-track approach. For the policy fairness pathway: institutions should ensure that information about preferential policies is proactively, transparently, and consistently communicated; procedural transparency in scholarship allocation and admissions should be explicitly documented and accessible. For the ethnic identity pathway: universities should invest in ethnic cultural programming—cultural festivals, heritage language courses, mentorship by senior minority students—that affirms the value of minority cultural identity within a national community frame. These two tracks are mutually reinforcing: students who perceive their cultural identity as recognized by institutional policy are more likely to perceive both the policies and the institution as fair.

The absence of a direct effect of policy awareness on national community identification implies that communication effectiveness cannot be judged by awareness metrics alone. Policy communication should be designed not merely to inform students about the existence of preferential policies, but to convey the institutional rationale behind them, the equity values they embody, and their recognition of the worth of ethnic cultural diversity. These are the psychological levers through which awareness becomes identification.

The moderation by interethnic contact suggests that structured programs promoting deep cross-ethnic interaction on campus—such as mixed residential arrangements, collaborative academic projects, and cross-cultural dialogue activities—may function as an independent pathway to national community belonging that supplements and, under certain conditions, partially substitutes for the ethnic identity route. Campus administrators would be well advised to treat high-quality interethnic contact as a distinct institutional tool in national identity education.

Finally, the heterogeneity findings argue for more differentiated communication strategies. Male students appear more responsive to the institutional cognition stage (awareness to fairness perception), suggesting that clear and informative policy communication may be particularly consequential for this group. Students raised outside Southwest China show attenuated responsiveness across both pathways, implying that institutions in non-minority-concentrated regions may need to invest more actively in both fairness-oriented policy communication and ethnic cultural programming to achieve comparable identification outcomes. Transparent, accessible, and consistently implemented policy information remains important across all groups, but the emphasis and delivery mode may need to be calibrated to regional and individual contexts.

### 4.4 Limitations and future directions

Several limitations should be noted. First, the cross-sectional design precludes causal inference; longitudinal and experimental designs are needed to establish the directional relationships proposed by the model. Second, the online sample exhibits a pronounced gender imbalance, with 68.3% female participants; the gender skew may limit the generalizability of the heterogeneity findings, and future research should seek more gender-balanced samples. Third, formal measurement invariance testing was not conducted prior to the multi-group SEM analyses. Configural invariance analyses confirmed that the five-factor structure is recoverable across subgroups (gender: male CFI = 0.890, female CFI = 0.930; region: Southwest CFI = 0.830, non-Southwest CFI = 0.900), but the absence of metric and scalar invariance tests means that between-group comparisons of path coefficients should be interpreted with caution. All multi-group findings are therefore presented as exploratory (Chen, 2007). Fourth, all five constructs exhibited pronounced negative skewness (range: −1.39 to −2.44), reflecting a substantial ceiling effect, particularly for the SCNC scale (*M* = 4.62, SD = 0.32 on a 5-point scale). This restricted variance likely attenuates observed path coefficients and may reduce the statistical power available to detect moderation effects, including the index of moderated mediation. Fifth, the AVE values for PFP (0.436), EI (0.397), and SCNC (0.368) fall below the conventional 0.50 threshold, indicating that a meaningful proportion of item variance is not captured by the latent constructs. This reflects the combined influence of construct multidimensionality and ceiling effects. While composite reliability values remained adequate and the five-factor structure was consistently supported over alternative models, the low AVE values represent a genuine measurement limitation; future research should employ refined scales with broader response formats to improve convergent validity. Additionally, the use of the heterotrait-monotrait ratio of correlations (HTMT) as a supplementary discriminant validity criterion is recommended, as it provides a more conservative estimate than the Fornell–Larcker criterion. Sixth, all five constructs relied on single-source self-report data, and while two CMB screening procedures were employed, these provide only preliminary reassurance; future research should employ multi-source designs or behavioral outcomes. Finally, other theoretically plausible mediating mechanisms—such as political efficacy, cultural confidence, and perceived social support—were not examined and may account for additional variance.

## 5 Conclusion

Ethnic education policy awareness is associated with the sense of Chinese national community among ethnic minority college students not directly, but through two parallel psychological channels of comparable magnitude. Through a policy fairness pathway, policy awareness is linked to perceptions of policy fairness that in turn are associated with stronger national community identification; through a social identity pathway, it is linked to ethnic identity, which is subsequently associated with superordinate national belonging. A negative interaction between ethnic identity and interethnic contact depth was observed at the path level, consistent with recategorization theory, though the overall moderated mediation effect did not reach statistical significance and should be treated as a preliminary exploratory finding. These findings advance a more nuanced understanding of the psychological mechanisms linking ethnic education policy to identification outcomes and offer evidence-based guidance for improving the effectiveness of ethnic education policy communication in Chinese higher education.

## Data Availability

The datasets generated and analyzed in this study are not publicly available because the participants’ informed consent did not include the public archiving of individual-level survey data. De-identified data supporting the findings of this study are available from the corresponding author upon reasonable request, subject to applicable institutional requirements.
