# Supplementary material for: Ethnic education policy awareness and national community identity among ethnic minority college students in China: the dual mediating roles of policy fairness perception and ethnic identity
Source: Front Psychol. 2026 Aug 11;17:1862762. doi: 10.3389/fpsyg.2026.1862762 (PMC13503368; doi:10.3389/fpsyg.2026.1862762)
Supplement: Supplementary file 1 [file Supplementary_file_1.docx]

**Appendix A**

**Complete Item Wording for All Scale Items (English and Chinese)**

**PA**

**PA1.** I am familiar with the main content of the state’s preferential education policies for ethnic minority students. [我了解国家针对少数民族学生实施的优惠教育政策的主要内容。]

**PA2.** I have a clear understanding of the specific support measures these preferential education policies provide for ethnic minority students. [我清楚这些优惠教育政策对少数民族学生的具体帮扶措施。]

**PA3.** I know how to access educational support for ethnic minority students through the relevant policy channels. [我知道如何通过相关政策渠道获得针对少数民族学生的教育支持。]

**PFP**

**PFP1.** I believe that information about education policies is publicly disclosed and transparent in the admission process. [在招生过程中，我认为教育政策的信息是公开且透明的。]

**PFP2.** The allocation of educational resources is fair to both ethnic minority and non-minority students. [教育资源的分配方式对少数民族学生和非少数民族学生来说都是公平的。]

**PFP3.** I believe that preferential policies have been implemented with adequate consideration of the differential needs of different ethnic groups, without generating fairness controversies. [我相信优惠政策在执行中，已经充分考虑了不同民族之间的差异性需求，未引发任何公平性争议。]

**EI**

**EI1.** I am proud of my ethnic group’s history and traditions (e.g., festivals and customs). [我为本民族的历史与传统（如节庆或风俗）而自豪。]

**EI2.** When studying or living away from home, I feel a special sense of warmth upon hearing my ethnic language or encountering traditional cultural elements. [当我在外地求学或生活时，听到本民族语言或看到传统文化元素，我会感到特别亲切。]

**EI3.** I actively participate in my ethnic group’s traditional activities (e.g., festivals and customs) in daily life. [我在日常生活中会积极参与本民族的传统活动（如节庆或风俗）。]

**EI4.** I believe that my ethnic group’s language, festivals, and customs have an important influence on my understanding of ethnic culture. [我认为本民族的语言、节日和习俗对我理解本民族文化有重要影响。]

**EI5.** I believe that my ethnic group’s cultural traditions enable me to understand my ethnic values more deeply. [我认为本民族的文化传统能让我更深入地理解了本民族价价值观。]

**IEC**

**IEC1.** I frequently collaborate with students from other ethnic groups on team tasks or community activities (e.g., volunteer services). [我经常与其他民族同学合作完成团队任务或参与公益活动（如志愿服务）。]

**IEC2.** When interacting with students from other ethnic groups, I proactively seek to understand their cultural backgrounds and lifestyles. [与其他民族同学互动时，我愿意主动了解他们的文化背景和生活习惯。]

**IEC3.** When misunderstandings arise from cultural differences, I actively communicate and work to find mutually acceptable solutions. [当因文化差异产生误解时，我会积极沟通并努力找到共同的解决方法。]

**SCNC**

**SCNC1.** I feel deeply proud to be a member of the Chinese national community. [我为自己是中华民族大家庭中的一员感到深深的自豪。]

**SCNC2.** When I hear of achievements by other ethnic groups in cultural or social domains, I feel proud, as these achievements are part of the collective progress of the Chinese nation. [当听到其他民族在文化或社会领域的成就时，我会感到自豪，因为这些成就是中华民族共同进步的一部分。]

**SCNC3.** I endorse the values of solidarity, inclusiveness, and mutual assistance advocated by the Chinese national community, and am willing to incorporate them into my daily conduct. [我认可中华民族整体倡导的团结、包容、互助等价値，并愿意将其融入自己的日常行为中。]

**SCNC4.** I believe that respecting multicultural diversity and ethnic differences is key to the solidarity of the Chinese national community in promoting social progress. [我认为在促进社会进步的过程中，尊重多元文化和民族差异是中华民族共同体团结的关键。]
